# Folic Acid and Pregnancy: Assessing Awareness, Timing, and Use of Folic Acid Among Pregnant Women in Medinah, Kingdom of Saudi Arabia

**DOI:** 10.7759/cureus.98709

**Published:** 2025-12-08

**Authors:** Abdulhakim M Alhazmi, Mohammed Elmuttalut

**Affiliations:** 1 Department of Clinical Pharmacy, Al-Rayan National Colleges, Medinah, SAU; 2 College of Medicine, Al-Rayan National Colleges, Medinah, SAU

**Keywords:** antenatal care, awareness, folic acid, neural tube defects, pregnancy

## Abstract

Background: Folic acid (FA) plays a crucial role in the prevention of neural tube defects (NTDs) and other congenital anomalies during fetal development. Despite the known benefits, awareness and proper utilization of FA among pregnant women remain inconsistent globally.

Objective: The overall objective of this study was to assess the awareness and practices related to FA use during pregnancy among Saudi women attending antenatal care clinics in Medinah, Saudi Arabia.

Methods: A cross-sectional study was conducted from October to December 2024 at selected antenatal clinics across the primary healthcare centers and the maternity hospitals in Medinah. A total of 400 Saudi pregnant women were recruited using a cluster sampling technique. Data were collected using a structured, validated questionnaire administered via face-to-face interviews. Descriptive statistics, Mann-Whitney Z-test, and Kruskal-Wallis H-test were used to analyze the data with IBM SPSS Statistics for Windows, version 26 (IBM Corp., Armonk, New York, United States). A p-value of <0.05 was considered statistically significant.

Results: Among the 400 participants, 194 (48.5%) were aged 31-40 years, 182 (45.5%) held a bachelor’s degree, and 306 (76.5%) were housewives. A total of 394 (98.5%) reported using FA during pregnancy, but only 76 (19.0%) correctly identified it as a vitamin. Additionally, 215 (53.8%) recognized its role in preventing neural tube defects. Awareness regarding the appropriate timing of FA intake was 283 (70.8%) for the first trimester and 175 (43.8%) for starting three months before pregnancy.

Conclusion: Although most pregnant women reported taking FA, there were notable gaps in their knowledge regarding its classification and proper timing of intake. These findings indicated the need for targeted educational campaigns through tailored health education programs, social platforms, and community-based programs to enhance awareness toward the importance of taking FA before and during pregnancy.

## Introduction

Folic acid (FA), a synthetic form of folate (vitamin B9), is essential for DNA synthesis, cellular growth, and embryonic neural development. Adequate maternal intake of FA before conception and during the first trimester of pregnancy significantly reduces the risk of neural tube defects (NTDs), such as spina bifida and anencephaly [1,2]. Despite global awareness of its benefits, many women of reproductive age lack adequate knowledge or fail to adhere to the recommended supplementation guidelines, resulting in preventable congenital anomalies [3,4].

Globally, approximately 300,000 newborns are affected by NTDs annually, with a disproportionate burden in low- and middle-income countries [5,6]. In high-income countries like the United States, mandatory FA fortification has contributed to a reduction in NTD prevalence to about five cases per 10,000 live births [7,8]. In contrast, countries in the Middle East and North Africa (MENA) region report considerably higher rates of 7-20 per 10,000 births [9,10]. Although the Saudi Ministry of Health has endorsed the use of FA before and during early pregnancy, several studies have reported inconsistent awareness and low levels of appropriate intake among Saudi women [11,12].

Existing literature highlights significant knowledge and practice gaps. For instance, Alreshidi et al. (2018) found that while 80% of women attending King Fahad Medical City outpatient clinics were aware that FA prevents birth defects, only 47% were taking the supplement at the appropriate time [9]. Another study conducted by Kumari et al. (2024) in India revealed that although 74% of women used iron and FA supplements during pregnancy, only 38.5% were aware of their purpose, and 16% knew the correct timing for supplementation [13]. These findings reflect an international concern, with regional variations influenced by cultural norms, healthcare access, and education levels [14,15]. Moreover, a study in Bahrain indicated that even among healthcare workers, knowledge gaps about essential preventive measures like FA intake persist [16].

Despite these insights, limited data exist from peripheral regions of Saudi Arabia, including Medinah. Most prior research has focused on central urban centers such as Riyadh, thereby resulting in variation in awareness that may affect health behavior and awareness. Additionally, few studies have systematically examined how socio-demographic variables such as income, employment, education, and reproductive history influence FA awareness and consumption patterns [12].

As congenital anomalies continue to pose a public health burden, particularly those preventable through proper micronutrient supplementation, localized evidence is critical for designing effective interventions [7,8]. The aim of this study was to assess the awareness, knowledge, and practices regarding FA supplementation during pregnancy among Saudi women in Medinah and to identify socio-demographic predictors of awareness levels.

The significance of this research lies in its potential to inform public health initiatives and maternal health education programs. By identifying gaps in knowledge and practice, the findings of this study may guide the development of targeted educational strategies tailored to the needs of Medinah region communities and contribute to reducing the prevalence of NTDs and improving pregnancy outcomes in Saudi Arabia and comparable settings.

## Materials and methods

This was a cross-sectional study conducted at antenatal care clinics within selected healthcare facilities in Medinah, Saudi Arabia, between October and December 2024. These included primary healthcare centers (PHCs) and maternity hospitals under the supervision of the Ministry of Health (MOH). The primary aim of the study was to assess awareness and practices related to FA supplementation among pregnant women.

Ethical approval was obtained from the Institutional Review Board, General Directorate of Health Affairs in Medinah (IRB Log No. 24-104). All participants signed a written informed consent form before enrollment. Data confidentiality and participant anonymity were strictly maintained. Research data was stored securely in a password-protected laptop for a minimum of three years, in compliance with institutional ethical guidelines.

Study population and sampling technique

The study included a total of 400 pregnant Saudi women. Participants were selected using a cluster sampling technique, targeting healthcare facilities that provide antenatal care services. The required sample size was calculated using Epi Info software version 7.2.5.0 (Centers for Disease Control and Prevention, Atlanta, Georgia, United States), with assumptions of a 95% confidence level (CI) and a 5% margin of error. Participants were eligible for inclusion if they were pregnant women attending antenatal care clinics during the study period, held Saudi nationality, and agreed to participate by providing written informed consent. Women were excluded if they refused to participate, declined to sign the consent form, or did not meet the inclusion criteria, such as being non-Saudi nationals.

Data collection

Data were collected through face-to-face structured interviews using a validated questionnaire specifically developed for this study (see Appendices). The questionnaire was divided into three main sections. The first section captured demographic information, including age, marital status, educational level, employment status, and monthly income. The second section focused on medical and obstetric history, such as the presence of chronic diseases, history of miscarriage, number of previous pregnancies, and whether the participant had children with special needs. The third section assessed awareness of FA through a questionnaire that addressed the identity of FA, its preventive role in neural tube defects, and the appropriate timing for its intake. Each awareness item was coded as correct (1) or incorrect (0), with the total score used to categorize the level of awareness.

Statistical analysis

Data were entered and analyzed using IBM SPSS Statistics for Windows, Version 26 (IBM Corp., Armonk, New York, United States). Descriptive statistics were used to summarize the data: frequencies and percentages for categorical variables, and mean ± standard deviation (SD) for continuous variables. Normality of the awareness score distribution was assessed using the Kolmogorov-Smirnov test. Since the awareness scores were not normally distributed, non-parametric tests were applied. Differences in awareness scores across sociodemographic variables were analyzed using the Mann-Whitney U-test and the Kruskal-Wallis H-test. A p-value < 0.05 was considered statistically significant*.*

## Results

A total of 400 Saudi pregnant women were recruited for this study. Table 1 summarizes their socio-demographic characteristics. Nearly half of the participants were between 31-40 years of age (n=194, 48.5%), and the vast majority were married (n=394, 98.5%). Regarding education, 182 (45.5%) held a bachelor’s degree. Most respondents were housewives (n=306, 76.5%), and slightly more than half reported a monthly household income of more than 5,000 SAR (n=202, 50.5%). Chronic medical conditions were reported by 62 (15.5%) participants. Additionally, 90 (22.5%) were experiencing their first pregnancy, and 177 (44.3%) had three or more children. Only 11 (2.8%) reported having a child with special needs or a hereditary disorder, while 170 (42.5%) had a history of miscarriage.

**Table 1:** Socio-demographic characteristics of study participants (N=400)

Study variables	Frequency (Percentage)
Age group	-
15 – 20 years	2 (0.50%)
21 – 30 years	160 (40.0%)
31 – 40 years	194 (48.5%)
>40 years	44 (11.0%)
Marital status	-
Married	394 (98.5%)
Divorced	4 (01.0%)
Widowed	2 (0.50%)
Educational level	-
No degree	52 (13.0%)
High school graduate or diploma	155 (38.8%)
Bachelor's degree	182 (45.5%)
Postgraduate	11 (02.8%)
Occupation	-
Student	19 (04.8%)
Housewife	306 (76.5%)
Healthcare Provider	15 (03.8%)
Employee	60 (15.0%)
Monthly income (SAR)	-
<5,000	198 (49.5%)
5,000 – 10,000	113 (28.2%)
>10,000	89 (22.3%)
Do you have any chronic disease? (hypertension, Diabetes, asthma or other)	-
Yes	62 (15.5%)
No	338 (84.5%)
Is this your first pregnancy?	-
Yes	90 (22.5%)
No	310 (77.5%)
How many children do you have?	-
None	80 (20.0%)
One	63 (15.8%)
Two	80 (20.0%)
Three or more	177 (44.3%)
Have you had a child with special needs? Hereditary disease?	-
Yes	11 (02.8%)
No	389 (97.3%)
Have you experienced a miscarriage?	-
Yes	170 (42.5%)
No	230 (57.5%)

Table 2 showed that only 76 (19.0%) correctly identified FA as a vitamin, while 215 (53.8%) believed that FA prevents NTDs. Additionally, 175 (43.8%) reported that FA should be taken three months before pregnancy, while 283 (70.8%) indicated that the first trimester is the most appropriate time to take FA.

**Table 2:** Awareness about folic acid identity, purpose, and timing

Awareness about folic acid	Frequency (Percentage)
What do you think folic acid is? (vitamin)	76 (19.0%)
What do you think folic acid does? (prevent neural tube defects)	215 (53.8%)
What is the most appropriate time to take folic acid?	-
Three months before pregnancy	175 (43.8%)
First trimester	283 (70.8%)

**Figure 1:**
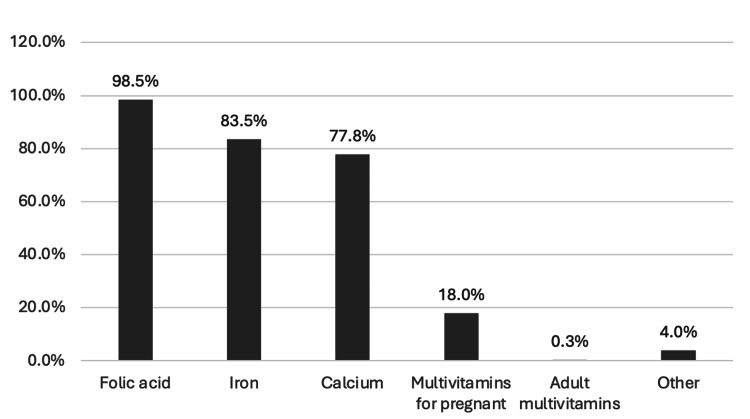
Use of vitamins and minerals during pregnancy

Figure 1 indicates that the most commonly used vitamins and minerals during pregnancy were FA (n=394, 98.5%), followed by iron (n=334, 83.5%) and calcium (n=311, 77.8%).

Table 3 showed that the awareness scores among the socio-demographic characteristics of the women indicated that higher awareness scores were associated with having higher education (Z=3.317; p=0.001), being employed/student (Z=1.916; p=0.028), increasing monthly income (Z=3.489; p<0.001), and no previous history of miscarriage (Z=2.375; p=0.018). No significant differences were observed between awareness scores in terms of age, associated chronic disease, first pregnancy, number of children, and having a child with special needs (p>0.05).

**Table 3:** Differences in awareness score based on the socio-demographic characteristics of the study participants (N=400) § P-value has been calculated using Mann-Whitney Z-test. ‡ P-value has been calculated using Kruskal Wallis H-test. ** Significant at p<0.05 level.

Factor	Awareness Score (4) Mean ± SD	Z-test	P-value ^§^
Age group			
≤30 years	1.78 ± 0.88	1.788	0.074
>30 years	1.93 ± 0.81		
Educational level			
Diploma or below	1.75 ± 0.82	3.044	0.002 **
Bachelor or higher	2.01 ± 0.85		
Occupation			
Employed/Student	2.09 ± 0.85	2.864	0.004 **
Housewife	1.81 ± 0.83		
Monthly income (SAR)			
<5,000	1.82 ± 0.86	1.181	0.238
≥5,000	1.93 ± 0.83		
Associated chronic disease			
Yes	1.94 ± 0.90	0.604	0.546
No	1.86 ± 0.83		
Is this your first pregnancy?			
Yes	1.79 ± 0.84	0.985	0.325
No	1.89 ± 0.85		
How many children do you have? ^‡^			
None	1.81 ± 0.86	0.253	0.615
1 - 2	1.92 ± 0.88		
≥3	1.86 ± 0.81		
Have you had a child with special needs? Hereditary disease?			
Yes	1.82 ± 0.87	0.010	0.992
No	1.87 ± 0.84		
Have you experienced a miscarriage?			
Yes	1.91 ± 0.81	0.727	0.467

## Discussion

This study provided a comprehensive assessment of awareness and practices related to FA among pregnant Saudi women in Medinah, revealing important insights into knowledge levels, socio-demographic influences, and supplement use. While the majority of participants reported taking FA during pregnancy (98.5%), a substantial proportion of participants demonstrated limited understanding of its identity (only 19.0% correctly identified it as a vitamin), benefits (53.8% recognized its role in preventing neural tube defects), and optimal timing (43.8% knew it should be taken three months before pregnancy), indicating the gap between knowledge and practice.

One of the key findings in this study was that only 19% of respondents correctly identified FA as a vitamin. This indicates a low level of basic factual knowledge, despite nearly universal supplement use. A similar knowledge gap was reported in a study conducted by Kumari et al. (2024), where only 38.5% of pregnant women knew the purpose of taking FA, despite 74% reporting its use. This suggests that supplementation may be routine or provider-prescribed rather than informed by personal understanding. In contrast, a study by Alreshidi et al. (2018) found that 80% of women were aware that FA prevents birth defects, although only 47% were taking it correctly, indicating better knowledge but poorer adherence [9].

Regarding the function of FA, 53.8% of the participants in this study correctly recognized its role in preventing NTDs. This finding is slightly lower than the 80% awareness reported by Alreshidi et al. (2018) in Riyadh [9] but higher than the 38.5% reported by Kumari et al. (2024) in India [13]. The moderate level of awareness observed in the current study may reflect region-specific variation in health education and antenatal counseling services. It also reflected the need for strengthening health promotion and education strategies in peripheral cities, compared to central urban centers.

Awareness regarding the appropriate timing of FA intake was mixed: 43.8% identified the correct recommendation of starting three months before pregnancy, and 70.8% knew that it should be taken during the first trimester. These findings were consistent with the results from Alreshidi et al., where 42.2% of women correctly identified the timing [9]. However, they are markedly higher than those reported in the study by Kumari et al., where only 16% knew the recommended duration of FA supplementation [13]. These comparisons suggested that although awareness of FA timing remains inadequate globally, Saudi women demonstrate relatively better knowledge, possibly due to antenatal care protocols followed in the Kingdom's healthcare system.

Socio-demographic analysis revealed several significant associations with FA awareness. Women with a bachelor’s degree or higher had significantly higher awareness scores (p = 0.001). Similar observations from multiple studies indicated that higher education levels correlate with improved health literacy and preventive practices [11,16]. Education likely enhances the capacity to comprehend medical advice and to seek reliable health information, contributing to improved knowledge and behaviors during pregnancy.

Employment status was also significantly associated with awareness, with employed women and students showing better awareness than housewives (p = 0.028). This finding aligned with Sowar et al.'s study findings, indicating that working women tend to have greater exposure to health education through workplace programs, social networks, and greater access to digital resources [11]. In contrast, housewives may have limited access to health information beyond what is provided during clinical visits, which may be brief or inconsistently delivered.

Other factors such as age group, presence of chronic diseases, number of children, first pregnancy status, and having a child with special needs were not significantly associated with FA awareness. These findings were consistent with the study by Sowar et al. [11], which found no significant association between parity or chronic illness and knowledge of preventive health behaviors, suggesting that these variables may be less influential in shaping awareness about FA compared to education and socioeconomic factors.

Finally, the high rate of reported FA use (98.5%) in this study is noteworthy and encouraging. It exceeds rates reported in similar studies. For example, Alreshidi et al. reported 47% usage [9], while Kumari et al. reported 74% [13]. This high rate may reflect improved compliance due to public health campaigns, routine supplementation policies in government clinics, or adherence to provider recommendations regardless of personal knowledge. However, the mismatch between high usage and low knowledge suggests a reliance on provider-initiated supplementation rather than informed maternal decisions. This emphasized the need not only to ensure the provision of supplements but also the provision of a health education program to enhance understanding of their purpose and correct usage.

## Conclusions

This study highlighted a notable discrepancy between the high rate of FA use during pregnancy and the limited awareness among participants regarding its identity, purpose, and optimal timing. Awareness was significantly associated with higher educational attainment, employment status, and higher household income, suggesting that socio-demographic factors play a crucial role in shaping maternal health literacy. Also, the findings highlighted the importance of integrating structured health education into routine antenatal care services to ensure that FA supplementation is not just a passive clinical practice but an informed maternal choice. Addressing knowledge gaps through targeted educational interventions, especially for women with lower educational levels or socioeconomic status, could enhance adherence to evidence-based supplementation guidelines and contribute to the prevention of birth defects. Future research should explore the underlying barriers to knowledge acquisition among specific subgroups and evaluate the effectiveness of health education strategies in improving FA awareness and use.

## Appendices

Study questionnaire

To download the questionnaire, please click on the following link: https://we.tl/t-JbxX0RwBYA
